# miRNA Differential Expression Profile Analysis and Identification of Potential Key Genes in Active Tuberculosis

**DOI:** 10.1111/jcmm.70567

**Published:** 2025-05-19

**Authors:** Jun Yang, Hanliang Dan, Yeng Chen, Linrong Zou, Shanshan Liu, Feng Wang, Maslinda Binti Musa

**Affiliations:** ^1^ Faculty of Applied Sciences Universiti Teknologi MARA Shah Alam Selangor Malaysia; ^2^ Department of Laboratory Medicine Guilin Medical University Affiliated Hospital Guilin Guangxi China; ^3^ Nanxishan Hospital of Guangxi Zhuang Autonomous Region (The Second People's Hospital of Guangxi Zhuang Autonomous Region) Guilin Guangxi China; ^4^ Faculty of Dentistry University of Malaiya Kuala Lumpur Malaysia

**Keywords:** active tuberculosis, differential expression, gene regulation, microRNA

## Abstract

*Tuberculosis* (TB), caused by *Mycobacterium* TB (MTB), remains a significant global health issue, particularly in developing nations. MicroRNAs (miRNAs) are non‐coding RNAs (ncRNAs) that modulate immune responses and play a pivotal role in the pathogenesis of MTB by altering host immune defences. Insights into the regulatory functions of these miRNAs have revealed mechanisms through which MTB evades immune surveillance and establishes persistent infections, highlighting the critical role of miRNA networks in TB pathogenesis. The purpose of this study was to analyse miRNA expression in plasma from TB patients, to predict target genes, and to construct regulatory networks to elucidate the roles of miRNAs in TB pathogenesis. Plasma samples from three patients with active TB and three healthy controls were analysed using high‐throughput small RNA sequencing. DEMs were identified using DESeq2, and target genes were predicted via TargetScan and miRWalk. Protein–protein interaction (PPI) networks were constructed using STRING and Cytoscape. Functional enrichment analyses were performed using Gene Ontology (GO) and KEGG databases. A total of 23 DEMs were identified, including 17 upregulated and 6 downregulated miRNAs. hsa‐miR‐15a‐5p emerged as the most significantly upregulated miRNA. PPI network analysis highlighted CCND1, CDK6 and CCND2 as central genes, potentially regulated by miR‐15a‐5p. GO and KEGG analyses revealed enrichment in pathways related to cell cycle regulation, kinase activity and protein complex formation, suggesting their involvement in TB pathogenesis. This study identifies hsa‐miR‐15a‐5p and its target genes as key components in the regulatory landscape of TB. These findings offer new insights into the molecular mechanisms of TB and propose potential biomarkers and therapeutic targets for future research.

## 1 Introduction

Tuberculosis (TB), caused by *Mycobacterium tuberculosis* (MTB), remains one of the most significant global health challenges, particularly in developing regions [1]. In 2022, Malaysia reported approximately 26,000 new TB cases, corresponding to a rate of 80 cases per 100,000 population [1, 2]. The disease disproportionately affects individuals with comorbidities such as diabetes, HIV and immunosuppressive conditions, as well as those living in crowded conditions [3]. According to the World Health Organisation, TB was responsible for approximately 1.3 million deaths in 2022, ranking as the second leading cause of death from infectious diseases after COVID‐19 [1]. Despite advancements in diagnostic tools and treatment regimens, challenges such as delayed diagnosis, the emergence of multidrug‐resistant strains, and the complexity of distinguishing between active, latent and cured infections hinder TB management [4]. These challenges underscore the need for a deeper understanding of TB pathogenesis and host‐pathogen interactions.

Upon infection, MTB initially encounters the host's innate immune system, which includes natural killer cells, γδ T cells and macrophages [1]. These immune cells play a pivotal role in recognising and eliminating the pathogen, while activating the adaptive immune response to combat MTB [5]. The delicate immune balance between the host and MTB largely determines the outcome of the infection, whether it results in successful pathogen clearance, latent infection or progression to active disease [6]. Although innate immune cells are crucial in the early stages of infection, the adaptive immune response, mediated by T cells and other components, further contributes to the control of MTB. However, MTB has evolved sophisticated mechanisms to evade host immunity and establish persistent infection, including the modulation of immune pathways.

Recent advances in molecular biology have elucidated the critical roles of microRNAs (miRNAs) in regulating immune responses during TB infection [7]. MiRNAs, small ncRNA molecules, influence gene expression post‐transcriptionally and are implicated in diverse biological processes including inflammation, immune regulation and cellular stress responses [8]. In the context of TB, miRNAs have emerged as key regulators of the immune balance between the host and MTB [9]. Studies have shown that miRNAs such as miR‐21, miR‐155 and miR‐146a are crucial in modulating macrophage activation, cytokine production, T cell differentiation and autophagy [10]. These processes are essential for controlling MTB infection and determining the disease outcome. For instance, miR‐21 and miR‐155 have been implicated in influencing macrophage polarisation, whereas miR‐146a regulates inflammatory responses, collectively facilitating MTB's evasion of host immunity and establishing persistent infection [11, 12].

Despite the growing body of evidence linking miRNAs to TB pathogenesis, significant knowledge gaps remain. Specifically, the miRNA‐mediated mechanisms distinguishing active TB from latent infection are not fully understood. Identifying DEMs in TB patients compared to HCs could uncover critical miRNA‐regulated pathways involved in TB progression and immune evasion. Such insights could provide valuable biomarkers for TB diagnosis and prognostic assessment, as well as potential therapeutic targets.

This study aimed to investigate the differential expression of miRNAs in TB patients and HCs to identify miRNA signatures associated with active TB. By exploring the miRNA‐mediated regulatory networks relevant to TB pathogenesis, this research seeks to enhance our understanding of TB immunopathology and contribute to the development of improved diagnostic and therapeutic strategies.

## 2 Materials and Methods

### 2.1 Sample Collection and Preparation

Samples were collected from October 2023 to January 2024, comprising three patients (S1, S2, S3) diagnosed clinically with TB at the Affiliated Hospital of Guilin Medical University. The group included two males and one female, averaging 36 years in age. Additionally, three individuals (S4, S5, S6) undergoing health check‐ups at the Health Examination Center of the Affiliated Hospital of Guilin Medical University were selected as the HC group during the same period. This group also consisted of two males and one female, with an average age of 36 years (Table 1). All six individuals (samples S1–S6) were unrelated and had no known familial or genetic relationships, ensuring sample independence and minimising genetic background bias in downstream analyses.

**TABLE 1:** Characteristics of study participants with active tuberculosis and HC.

Sample	Gender	Age (year)	Disease characteristics	TST	IGRA
S1	Female	28	Low‐grade fever, cough for 4 weeks, with typical radiological changes in the lungs	Positive	Positive
S2	Male	35	Low‐grade fever, cough for 5 weeks, with typical radiological changes in the lungs	Positive	Positive
S3	Male	46	Low‐grade fever, cough for 4 weeks, with typical radiological changes in the lungs	Positive	Positive
S4	Female	28	HC	Negative	Negative
S5	Male	36	HC	Negative	Negative
S6	Male	45	HC	Negative	Negative

Abbreviations: HC, Healthy Control Group; IGRA, Interferon‐γ Release Assay; TST, Tuberculosis Skin Test.

Active TB patients were diagnosed on the basis of clinical symptoms, bacterial culture and radiographic evidence. No medical treatment had been administered to the patients prior to blood extraction. TB patients were excluded if they had: co‐infections (e.g., pneumonia, COPD, lung cancer), prior anti‐TB treatment, recent vaccination (e.g., BCG within 3 months) HIV/AIDS, immunosuppressive therapy, pregnancy, lactation, organ transplantation or incomplete clinical/laboratory data. Healthy individuals were confirmed to have no infections, no family history of hereditary diseases, and no impaired immune function.

The study received approval from the local ethics committee (Guilin Medical University Affiliated Hospital). Written informed consent was obtained from all participants before enrolment.

Fresh whole‐blood samples (5 mL) were collected in EDTA‐coated anticoagulant tubes from both HCs and patients with active TB (ATB). Samples were immediately centrifuged at 4°C and 3000 *g* for 15 min to separate the plasma, which was then stored at −80°C until RNA extraction.

### 2.2 RNA Extraction and Quality Control

Total RNA was extracted using the Total RNA Extraction Kit (Thermo Fisher Scientific Inc., MA, USA), following the manufacturer's instructions. RNA quantity and quality were determined using the Nanodrop 2000 (Thermo Fisher Scientific, MA, USA) and Agilent 2100 Bioanalyzer systems (Agilent Technologies). Only samples with an RNA Integrity Number (RIN) above 7 were utilized to construct a high‐quality total RNA sequencing (RNA‐seq) library.

### 2.3 Small RNA Sequencing by Illumina MGI2000

Total RNA was extracted using the TRIzol reagent according to the manufacturer's protocol. For library preparation, small RNA (sRNA) libraries were constructed using the NEBNext Multiplex Small RNA Library Prep Set for Illumina (New England Biolabs, USA). Briefly, a 3′ SR adaptor was ligated to the small RNAs, followed by 5′ SR adaptor ligation. Reverse transcription was then performed to synthesise first‐strand cDNA. The resulting cDNA was amplified using PCR with indexed primers, enabling sample multiplexing.

PCR‐amplified cDNA fragments corresponding to small RNAs (typically 140–160 bp in length including adaptors) were purified by polyacrylamide gel electrophoresis (PAGE) to remove adapter dimers and undesired fragments. The final libraries were quantified using a Qubit Fluorometer (Thermo Fisher Scientific) and evaluated for quality and size distribution using an Agilent 2100 Bioanalyzer (Agilent Technologies, USA).

Library qualification criteria included: Clear single peak in Bioanalyzer electropherogram within the expected size range (140–160 bp);Absence of adapter dimers or abnormal peaks; Library concentration ≥ 10 nM.

Qualified libraries were pooled and sequenced on an Illumina HiSeq platform to generate 50 bp single‐end reads.

### 2.4 Data Analysis Quality Control and PCA Analysis

High‐quality clean data were obtained by processing fastq‐format data with Trimmomatic (v0.30), which included the following steps: (1) removing adapter sequences, (2) discarding 5′ or 3′ end bases containing Ns or with quality values below 20, (3) removing bases with an average quality score below 20 using a sliding window of 4 bp and (4) eliminating reads shorter than 18 bp after trimming.

Principal Component Analysis (PCA) was performed using the normalised miRNA expression data to assess sample clustering and variability between TB patients and healthy controls. The analysis was conducted in R using the ggplot2 and FactoMineR packages, and the first two principal components were used for visualisation.

### 2.5 miRNA Identification and Target Gene Prediction

miRNA identification and expression data were obtained using miRDeep2, a software tool for discovering known and novel miRNAs from deep sequencing data [13]. Website: https://github.com/rajewsky‐lab/mirdeep2/archive/v0.1.3.tar.gz. Differential expression analysis was conducted using the DESeq/DESeq2 Bioconductor package, based on the negative binomial distribution. Adjustments were made using Benjamini and Hochberg's method to control the false discovery rate, setting the adjust *p*‐value (*p*adj) threshold at < 0.05 for detecting DEMs. Target genes of miRNAs were predicted using TargetScan Human 8.0, miRWalk and the CTD database, with intersections determined by VENNY 2.1.0 [14, 15].

### 2.6 Construction of PPI Regulatory Network

The STRING database was utilised to search for known and predicted PPIs involving DEMs from TB patients and HCs. The study species were set as human (“ *Homo sapiens* ”), and free nodes were removed to construct the differential gene PPI network. The network was visualised using the CytoNCA plug‐in for network centrality analysis in Cytoscape 3.10.3 software.

### 2.7 Screening of Potential Key Genes

Potential key genes were predicted using cytoHubba, which ranks nodes based on network attributes. Three topological algorithms were employed to identify key genes in the interactome network, including degree centrality (DC), betweenness centrality (BC) and Maximal Clique Centrality (MCC). These algorithms calculated the importance or influence of nodes by DC, the degree of node interaction by BC, and the average shortest distance to other nodes in their connected components by MCC [16, 17].

### 2.8 GO and KEGG Enrichment Analysis

GOSeq (v1.34.1) was used to identify Gene Oncology (GO) terms for a list of miRNA target genes with significant adjusted *p*‐values less than 0.05. Additionally, topGO was utilised to plot the Directed Acyclic Graph (DAG).

Kyoto Encyclopedia of Genes and Genomes (KEGG) pathways were enriched for significantly differentially expressed genes using in‐house scripts.

### 2.9 Statistics

GraphPad Prism 8 software was used for graphing and statistical analysis. A *p*‐value of < 0.05 was considered statistically significant.

## 3 Results

### 3.1 Analysis of Quality and Length of High‐Throughput Sequencing Data

For the non‐TB group, 31,232,324 raw sequencing reads were obtained, each less than 51 bases in length. After adaptor and contaminant removal, 14,543,636 miRNA sequencing fragments, primarily between 21 and 22 nucleotides (nt) in length, were retained. In the TB group, 36,609,167 raw reads were processed to yield 16,823,394 miRNA sequencing fragments. The average GC content of the sequencing reads was 51%–53%, with Q30 values consistently above 95%. After filtering, the average GC content was 42%–50%, with Q30 values also above 95% (Tables 2 and 3).

**TABLE 2:** Sequencing raw data quality statistics.

Sample	Length	Reads	Bases	Q20 (%)	Q30 (%)	GC (%)	*N* (ppm)
S1	51	13,955,925	711,752,175	99.08	96.94	50.47	28.82
S2	51	10,922,922	557,069,022	98.99	96.71	51.93	15.77
S3	51	11,730,280	598,244,280	99.28	97.48	53.21	27.65
S4	51	10,312,667	525,946,017	98.99	97.06	51.88	27.31
S5	51	10,794,783	550,533,933	98.25	95.77	52.16	27.1
S6	51	10,124,874	516,368,574	99.17	97.29	52.48	27.66

**TABLE 3:** Data quality statistics after filtering of sequencing raw data.

Sample	Length	Reads	Bases	Q20 (%)	Q30 (%)	GC (%)	*N* (ppm)
S1	22.25	9,323,791	207,453,124	99.3	96.89	46.6	54.72
S2	22.02	2,338,891	51,493,443	98.78	95.74	42.44	25.79
S3	22.71	5,160,712	117,216,584	99.4	97.35	50.78	51.97
S4	22.22	4,588,862	101,956,103	99.19	96.8	47.93	51.63
S5	22.45	6,722,350	150,930,562	99.24	96.76	48.1	51.39
S6	22.66	3,232,424	73,253,888	99.27	97.07	50.33	52.75

*Note:* In the above table, sample is the name of sequencing samples, length is the average length, reads is the number of sequencing reads, and bases is the number of total bases; Q20 (%) and Q30 (%) are the percentages of bases with Phred values greater than 20 and 30 in the total number of bases, respectively; GC (%) is the percentage of the total number of bases in the total number of bases; *N* (ppm): the number of *N* per million bases. number of bases; *N* (ppm): the number of *N* per million bases.

### 3.2 PCA Analysis of miRNA Expression Profiles

**FIGURE 1:**
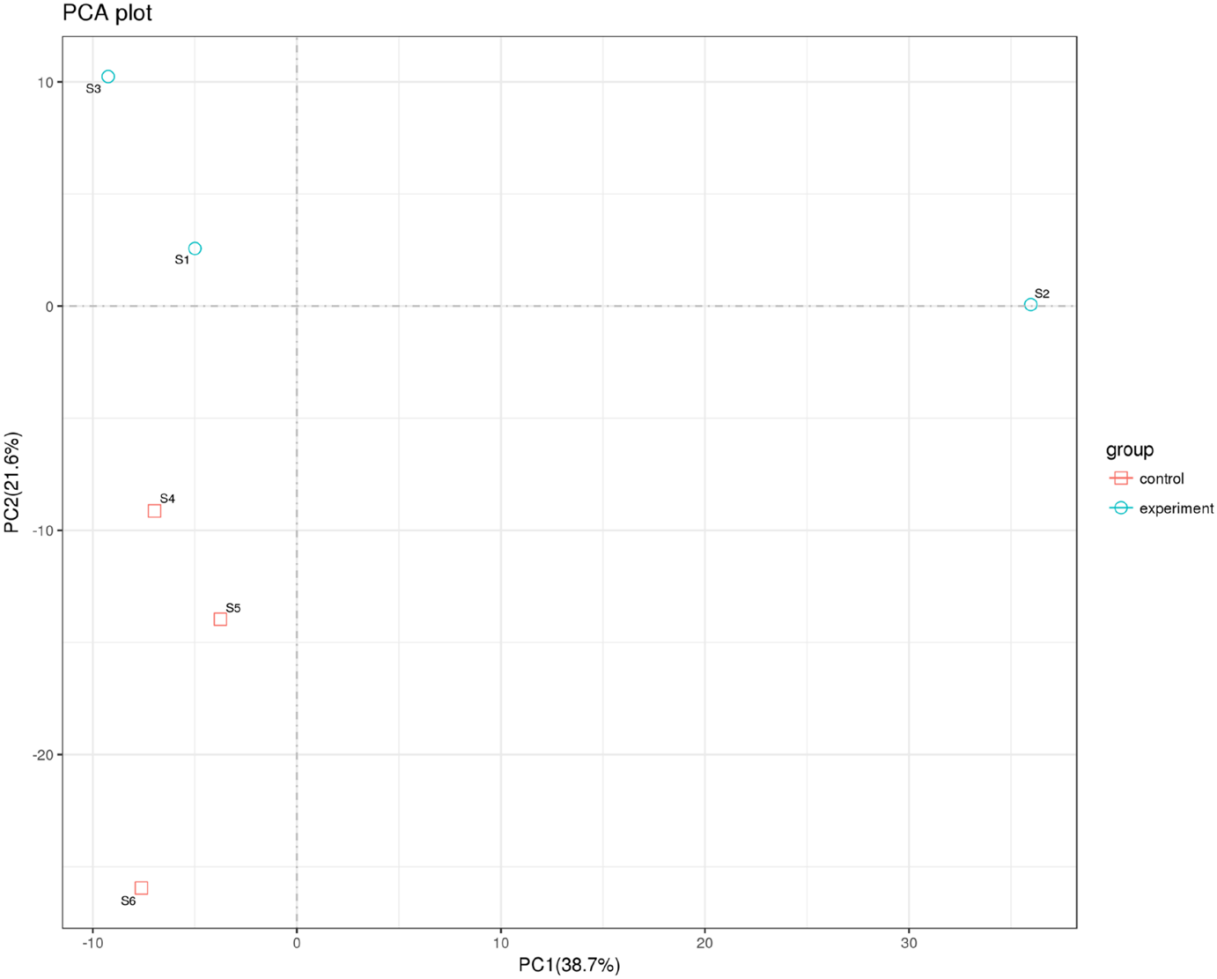
PCA plot of miRNA expression profiles in TB patients and healthy controls. Each point represents an individual sample. TB patient samples (experiment group) are shown as blue circles, and control samples as red squares.

To assess the global variation in miRNA expression between TB patients and healthy controls, Principal Component Analysis (PCA) was performed. As shown in Figure 1, the PCA plot demonstrates a clear separation between the experimental group (TB patients) and the control group (healthy individuals) along the first two principal components—PC1 (38.7%) and PC2 (21.6%). Samples from the same group clustered closely together, indicating good within‐group consistency, whereas distinct clustering between the two groups reflects substantial differences in miRNA expression profiles. This separation underscores the biological variability between TB and control samples and supports the robustness of the differential expression analysis.

### 3.3 Classification and Annotation of ncRNA

ncRNA, which does not encode proteins, includes various functional types such as rRNA, tRNA, snRNA, snoRNA and miRNA, in addition to RNAs of unknown function. All sequencing reads were aligned and annotated against these RNA types using sequence data from the Rfam database (version 14) with a sequence similarity of less than 90%. BLASTN software was employed for alignment, reporting only the best match for each sequence. The classification and annotation statistics for small RNA in both sample groups are detailed in Table 4.

**TABLE 4:** Small RNA classification annotation statistical results.

Sample	miRNA	rRNA	snRNA	snoRNA	tRNA	Others
S1	6,686,423	838,454	51,143	3667	88,365	830,277
S2	1,186,370	9494	2664	279	3268	961,994
S3	2,845,367	859,541	26,015	12,266	237,234	476,653
S4	2,776,853	339,578	71,839	27,773	119,698	560,541
S5	4,225,772	801,433	6406	5313	221,703	494,815
S6	1,693,063	398,740	3067	53,991	160,806	545,013

*Note:* S1–S3 were patient samples and S4–S6 were controls.

Potential miRNA precursors hairpin structures were analysed and scored using miRDeep2 to determine expression levels (Table 5), and miRNA prediction was conducted. “Known” miRNAs are those previously identified and recorded in the database, whereas “novel miRNA” are newly discovered during this study. In the TB group, 78,969 novel miRNAs were identified, compared to 98,995 in the non‐TB group.

**TABLE 5:** Statistics of miRNA expression levels.

Sample	known_miRNA	novel_miRNA
S1	7,775,467	53,045
S2	1,871,790	414
S3	3,379,896	25,510
S4	3,550,725	51,163
S5	5,033,522	39,573
S6	2,263,865	8259

*Note:* S1–S3 were patient samples and S4–S6 were controls.

### 3.4 Screening and Clustering Analysis of DEMs

**FIGURE 2:**
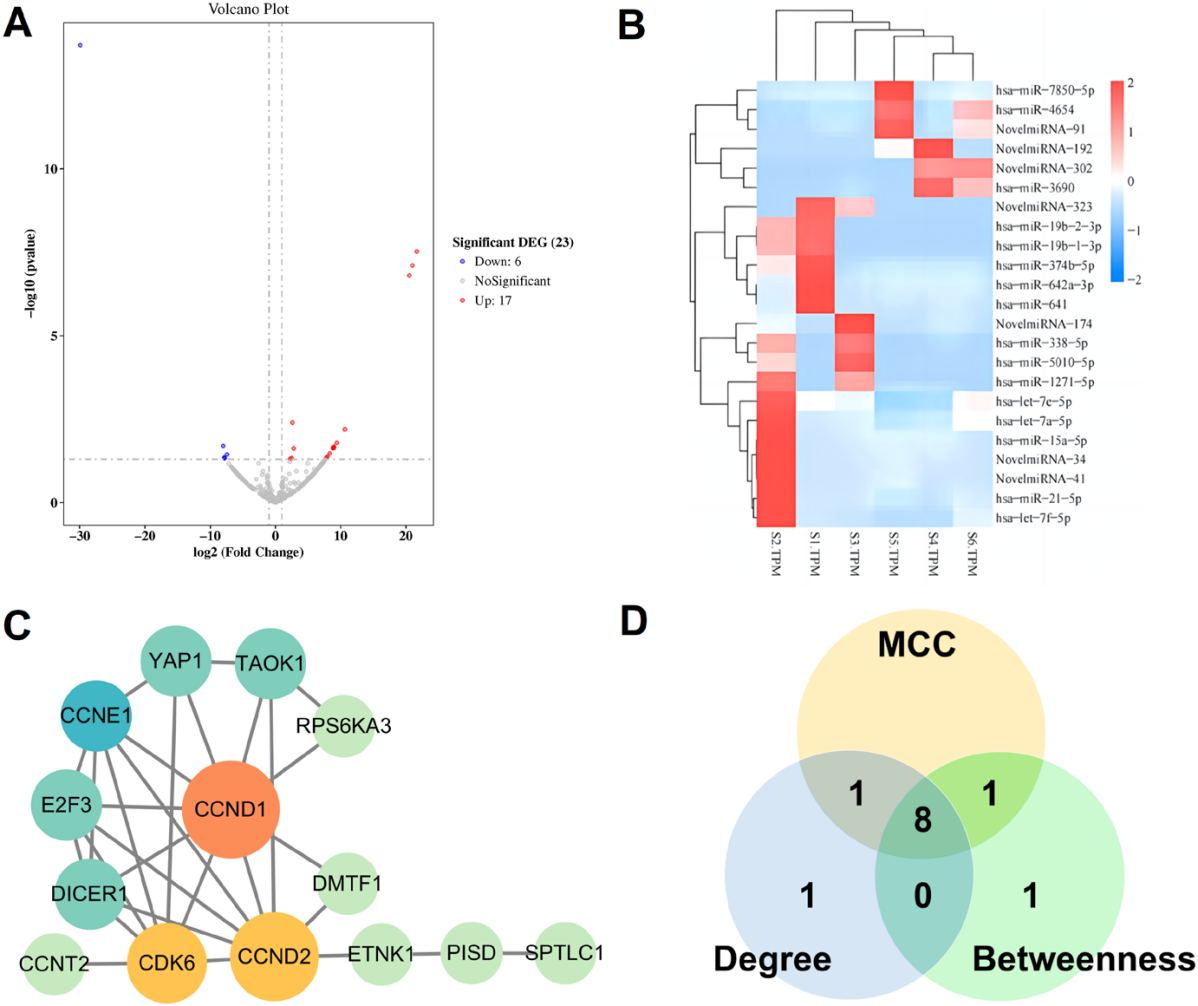
DEMs between samples: (A) Volcano plot. (B) Differential miRNA clustering heatmap (S1–S3 were patient samples and S4–S6 were controls). (C) PPI regulatory network diagram (The network's colour transitions from yellow to red, and node size increases from small to large. The redder and larger a node, the greater its degree; the greener and smaller a node, the lesser its degree). (D) Venn diagram showing the intersection of the top 10 key nodes by Degree, Betweenness and MCC algorithms. Yellow: Top 10 key nodes by MCC algorithm; Blue: Top 10 key nodes by Degree algorithm; Green: Top 10 key nodes by Betweenness algorithm.

miRNAs exhibiting a fold change greater than 2, *p*‐value ≤ 0.05 and identified as differentially expressed. Seventeen were upregulated, including NovelmiRNA‐34, hsa‐miR‐339‐5p, hsa‐miR‐15a‐5p, hsa‐miR‐21‐5p, hsa‐miR‐374b‐5p, hsa‐miR‐338‐5p, NovelmiRNA‐174, NovelmiRNA‐323, hsa‐miR‐19b‐2‐3p, hsa‐miR‐19b‐1‐3p, hsa‐miR‐642a‐3p, hsa‐let‐7f‐5p, hsa‐miR‐641, hsa‐miR‐1271‐5p, hsa‐miR‐5010‐5p, hsa‐let‐7e‐5p and hsa‐let‐7a‐5p. Six were downregulated: NovelmiRNA‐192, hsa‐miR‐7850‐5p, hsa‐miR‐4654, NovelmiRNA‐91, NovelmiRNA‐302 and hsa‐miR‐3690 (Table 6). Differential expression across samples was visualised using a volcano plot (Figure 2A).

**TABLE 6:** Spectrum of DEMs for up‐regulated expression.

DEMs	log_2_ (fold change)	*p*	*p*adj	Expression
NovelmiRNA‐34	21.66678301	2.97594E‐08	9.03E‐06	Up‐regulated
hsa‐miR‐339‐5p	21.00527571	7.79651E‐08	1.58E‐05	Up‐regulated
hsa‐miR‐15a‐5p	20.51474404	1.54869E‐07	2.35E‐05	Up‐regulated
hsa‐miR‐21‐5p	2.611901752	0.004006169	0.486349	Up‐regulated
hsa‐miR‐374b‐5p	10.67066179	0.00631366	0.546954	Up‐regulated
hsa‐miR‐338‐5p	9.402687097	0.016122437	0.546954	Up‐regulated
NovelmiRNA‐174	8.993820869	0.021374524	0.546954	Up‐regulated
NovelmiRNA‐323	8.895475971	0.022841113	0.546954	Up‐regulated
hsa‐miR‐19b‐2‐3p	8.894260642	0.022859772	0.546954	Up‐regulated
hsa‐miR‐19b‐1‐3p	8.894260642	0.022859772	0.546954	Up‐regulated
hsa‐miR‐642a‐3p	8.871272435	0.02321521	0.546954	Up‐regulated
hsa‐let‐7f‐5p	2.815659161	0.023689742	0.546954	Up‐regulated
hsa‐miR‐641	8.326357618	0.033165716	0.546954	Up‐regulated
hsa‐miR‐1271‐5p	8.003275201	0.040645231	0.546954	Up‐regulated
hsa‐miR‐5010‐5p	7.832585887	0.04514573	0.546954	Up‐regulated
hsa‐let‐7e‐5p	2.50023789	0.046871999	0.546954	Up‐regulated
hsa‐let‐7a‐5p	2.27837354	0.048861877	0.546954	Up‐regulated
NovelmiRNA‐192	−29.908165	1.93E‐14	1.17E‐11	Down‐regulated
hsa‐miR‐7850‐5p	−7.994968985	0.020218515	0.546954	Down‐regulated
hsa‐miR‐4654	−7.4095295	0.03613498	0.546954	Down‐regulated
NovelmiRNA‐91	−7.837613973	0.044924382	0.546954	Down‐regulated
NovelmiRNA‐302	−7.827396621	0.045205004	0.546954	Down‐regulated
hsa‐miR‐3690	−7.767749345	0.046872938	0.546954	Down‐regulated

*Note:* *p*‐value: Statistical significance values for differences. padj: Adjust *p*‐value by using Benjamini and Hochberg's method to control the false discovery rate (FDR).

Expression levels of DEMs were analysed, and clustering was performed using log_2_ TPM values. Blue signifies low‐expression miRNAs, whereas red indicates high‐expression miRNAs, with a colour gradient from blue to red reflecting increasing miRNA expression levels. The results indicated that hsa‐miR‐15a‐5p, hsa‐miR‐21‐5p, NovelmiRNA‐34, hsa‐miR‐339‐5p, hsa‐miR‐338‐5p and hsa‐let‐7f‐5p exhibited higher expression in the case group, whereas hsa‐miR‐7850‐5p, NovelmiRNA‐192 and hsa‐miR‐3690 showed higher expression in the control group (Figure 2B).

### 3.5 Prediction of DEMs Target Genes and Differential Gene PPI Network

The 23 DEMs were sorted in ascending order by padj‐value, and the top 4 miRNAs were selected: NovelmiRNA‐192 (low expression), NovelmiRNA‐34 (high expression), hsa‐miR‐339‐5p (high expression), hsa‐miR‐15a‐5p (high expression). TargetScan Human 8.0 and miRWalk were used to predict their downstream target genes, with intersections determined using VENNY 2.1.0, resulting in 96 target genes for expressed DEMs. By intersecting these genes with the target gene sets, 48 final target genes were identified. The 48 differentially expressed genes were entered into the STRING database, and free genes were removed to refine 27 significant genes. The PPI regulatory network was visualised using Cytoscape 3.10.1 software (Figure 2C), featuring 14 nodes and 29 edges.

### 3.6 Screening of Potential Key Genes of TB

Key genes from 14 differential genes in the PPI regulatory network were identified using three algorithms: Degree, Betweenness and MCC. The top 10 genes identified by each algorithm were designated as core genes (Table 7), and intersections were analysed using a Venn diagram (Figure 2D). CCND1 emerged as the most central gene across different algorithms, highlighting its potential role in TB pathogenesis (Table 8). CCND1, CDK6, CCNE1, CCND2, E2F3, DICER1, YAP1, TAOK1 were identified by the Degree, Betweenness and MCC ranks. CCND1 was predicted as a target gene of hsa‐miR‐15a‐5p by miRWalk on the basis of sequence and bind score.

**TABLE 7:** Top 10 differential genes ranked by degree, betweenness and MCC score.

Name	Degree	Name	Betweenness	Name	MCC
CCND1	9	CCND2	0.449	CCND1	134
CCND2	8	ETNK1	0.282	CDK6	127
CDK6	7	CCND1	0.207	CCNE1	126
CCNE1	6	CDK6	0.175	CCND2	125
DICER1	5	PISD	0.153	E2F3	120
E2F3	5	TAOK1	0.045	DICER1	120
TAOK1	4	CCNE1	0.021	YAP1	8
YAP1	4	YAP1	0.013	TAOK1	6
RPS6KA3	2	DICER1	0	PISD	2
DMTF1	2	E2F3	0	DMTF1	2

**TABLE 8:** Intersection data results of top 10 key nodes by Betweenness, MCC and Degree algorithms.

Name	Total	Elements
Betweeness and MCC and degree	8	CCND1, CDK6, CCND2, CCNE1, E2F3, DICER1, YAP1, TAOK1
MCC and degree	1	DMTF1
MCC and betweeness	1	PISD
Degree	1	RPS6KA3
Betweeness	1	ETNK1

### 3.7 GO Enrichment Analysis and KEGG Enrichment Analysis of Target Genes

**FIGURE 3:**
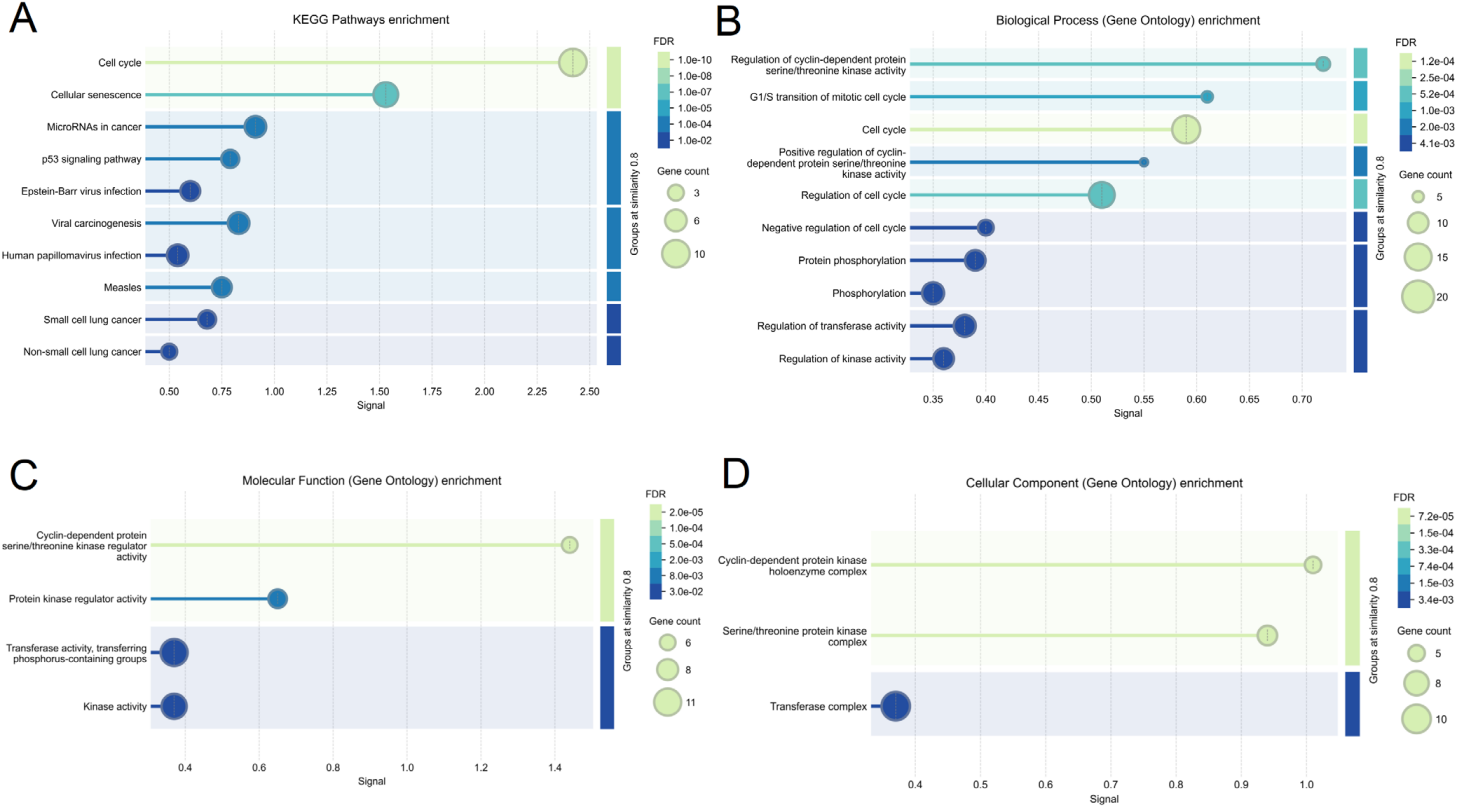
Differential miRNA target gene KEGG and GO enrichment bubble plot (The bubble size indicates the number of genes involved, and the colour gradient reflects the statistical significance, measured byFDR.). (A) KEGG Pathway enrichment; (B) GO Biological Process enrichment; (C) GO Molecular Function enrichment; (D) GO Cellular Component enrichment.

We conducted KEGG pathway and GO enrichment analyses on our 48 differentially expressed genes (DEGs) to identify key biological functions and pathways. In KEGG pathway enrichment, notably, the “Cell cycle” pathway exhibited the most significant enrichment (lowest FDR value) (Figure 3A), indicating a potential central role of cell cycle regulation in the biological processes underlying our dataset. Other enriched pathways include “Cellular senescence”, “MicroRNAs in cancer”, “p53 signaling pathway”, “Epstein‐Barr virus infection” and “Viral carcinogenesis”, suggesting that the DEGs are also involved in cancer‐related signalling, viral‐associated pathways and gene regulatory mechanisms.

To explore the functional roles of the differentially expressed genes (DEGs), Gene Ontology (GO) enrichment analysis was conducted across three categories: Biological Process (BP), Molecular Function (MF) and Cellular Component (CC). In the BP category (Figure 3B), significantly enriched terms included regulation of cyclin‐dependent protein serine/threonine kinase activity, G1/S transition of the mitotic cell cycle and positive regulation of the cell cycle, highlighting a strong association of DEGs with cell cycle progression and regulatory mechanisms. Within the MF category (Figure 3C), the DEGs were primarily enriched in cyclin‐dependent protein serine/threonine kinase regulator activity, protein kinase regulator activity and kinase activity, suggesting that key enzymatic and regulatory functions are involved in the observed biological processes.

In the CC analysis (Figure 3D), the most enriched terms were cyclin‐dependent protein kinase holoenzyme complex, serine/threonine protein kinase complex and transferase complex, indicating that these DEGs are largely localised to functional protein complexes involved in phosphorylation and signal transduction.

Overall, the GO enrichment analysis emphasises the involvement of DEGs in cell cycle regulation, kinase activity and protein complex formation, which may play critical roles in the underlying biological mechanisms of the studied condition.

## 4 Discussion

TB, a chronic infectious disease caused by MTB, poses a significant threat to human health [18]. After infection, MTB can manipulate the host's immune response through various mechanisms, including lysosomal phagocytosis, apoptosis, autophagy, inflammation and modulation of innate immune responses such as the expression of MHC class II molecules and MHC class I antigen presentation by dendritic cells. This enables the pathogen to persist within the human body for extended periods [4, 19].

miRNAs, non‐coding, single‐stranded RNA molecules approximately 18–25 nucleotides in length, inhibit the expression of target genes by binding to the 3′ untranslated region (3′ UTR) of target mRNAs [20]. Multiple miRNAs can regulate the same gene, influencing various biological processes through two main mechanisms [21, 22]: (i) mRNA degradation via cleavage, or (ii) inhibition of the mRNA translational process. In some cases, both mechanisms may occur simultaneously, allowing for precise regulation of numerous physiological and pathological processes [23]. Previous studies have demonstrated that several miRNAs, such as miR‐21, miR‐155, miR‐146a and miR‐21‐5p, modulate immune responses during TB infection [24, 25].

In the present study, high‐throughput sequencing of plasma samples from TB patients and healthy controls identified 23 differentially expressed miRNAs (DEMs), comprising 17 upregulated and 6 downregulated miRNAs. Among them, hsa‐miR‐15a‐5p was identified as the most significantly upregulated miRNA on the basis of adjusted *p*‐values, suggesting a potential biomarker and regulator role in TB pathogenesis.

As a member of the miR‐15/16 family, miR‐15a‐5p modulates various signalling pathways involved in cell cycle control, apoptosis and inflammatory cytokine production [26, 27]. Previous studies have shown that miR‐15a‐5p targets genes such as *BCL2*, *IKKα* and components of the NF‐κB signalling pathway, thereby modulating the balance between pro‐ and anti‐inflammatory responses [28]. Additionally, miR‐15a‐5p plays a role in macrophage activation and polarisation, influencing cytokine secretion and regulating autophagy and apoptosis—processes critical to host defence against TB [29].

Through these mechanisms, miR‐15a‐5p can impact macrophage‐mediated bacterial clearance and inflammatory regulation, making it a potential biomarker and therapeutic target in TB and other infectious diseases. Despite its regulatory potential, the role of miR‐15a‐5p in TB remains underexplored. A previous study by Cengiz et al. reported a downregulation of miR‐15a‐5p in pulmonary TB, which contrasts with our findings [30]. These discrepancies may reflect differences in infection stages or sample collection timing. Moreover, the diagnostic and prognostic value of miR‐15a‐5p in TB requires further validation in larger cohorts.

Target gene prediction using TargetScan and miRWalk identified 48 potential targets, refined to 27 genes through intersection with the CTD database. These genes were entered into the STRING database to construct the PPI regulatory network using Cytoscape 3.10.1 software, which contained 14 nodes and 27 edges. The top 10 genes identified by the intersection screening of these algorithms were considered potential diagnostic targets of TB. Interestingly, CCND1, CDK6 and CCND2 predicted targets of has‐miR‐15a‐5p, suggesting a potential post‐transcriptional regulatory axis by which miR‐15a‐5p may modulate host cellular responses in TB. These findings provide novel insights into the molecular mechanisms underlying TB pathogenesis and highlight key genes that may serve as diagnostic biomarkers or therapeutic targets. However, further experimental validation and functional assays are necessary to confirm the regulatory relationships and biological significance of these candidate genes in TB infection.

CCND1, CDK6 and CCND2 are critical regulators of the cell cycle, particularly in the transition from G1 to S phase [31]. CCND1 (Cyclin D1) and CCND2 (Cyclin D2) belong to the D‐type cyclin family, which form complexes with cyclin‐dependent kinases such as CDK6 to drive cell cycle progression [32]. These complexes phosphorylate the retinoblastoma (Rb) protein, leading to the release of E2F transcription factors and activation of genes required for DNA synthesis [33]. Beyond cell proliferation, emerging evidence suggests that D‐type cyclins and CDK6 also play roles in immune cell development, inflammatory responses and host‐pathogen interactions [34, 35]. In infectious diseases, dysregulation of these genes may influence macrophage activation, apoptosis and cellular immune defence mechanisms [36, 37]. Prior studies have also linked miR‐15a‐5p regulation of CCND1 and CDK6 to cancer biology, suggesting a broader relevance of this regulatory axis: Zhao found that the expression level of miR‐15a‐5p in pancreatic cancer was negatively correlated with CDK6 through bioinformatics analysis [38], whereas Li demonstrated that hsa‐miR‐15a‐5p alleviated the proliferation, migration and invasion of colon cancer by targeting the CCND1 gene [39].

The integrated KEGG and GO enrichment analyses provided valuable insights into the biological functions and regulatory mechanisms associated with the differentially expressed genes (DEGs) in this study. KEGG pathway analysis revealed that the cell cycle pathway was the most significantly enriched, suggesting that dysregulation of cell proliferation and cell cycle progression may play a central role in the underlying biological context. Additional enrichment in pathways such as cellular senescence, p53 signalling pathway and microRNAs in cancer further supports the involvement of tumour suppressor mechanisms, DNA damage responses and post‐transcriptional gene regulation in the studied condition. These pathways are well known for their crucial roles in controlling cell growth, genomic stability and oncogenesis, but they also regulate the cell cycle during the TB infection through phosphorylation of RB, regulation of inflammation and immune cell proliferation [40, 41, 42].

Consistent with the KEGG findings, GO enrichment analysis across three domains—Biological Process (BP), Molecular Function (MF) and Cellular Component (CC)—highlighted a predominant focus on cell cycle regulation and kinase‐mediated signalling. BP enrichment showed significant overrepresentation of terms related to regulation of cyclin‐dependent protein kinase activity, G1/S phase transition and positive regulation of the cell cycle, reinforcing the notion that altered cell cycle checkpoints and proliferation control are key features of the DEG profile [43]. MF analysis further revealed enrichment in cyclin‐dependent kinase regulator activity, kinase activity and transferase activity, underscoring the functional importance of enzymatic and signalling components in the observed molecular landscape. Meanwhile, CC terms such as cyclin‐dependent protein kinase holoenzyme complex and serine/threonine kinase complexes indicate that many DEGs are structurally localised to functional protein assemblies involved in intracellular signalling [44].

Collectively, these findings suggest that the DEGs are not only central to cell cycle regulation and proliferative control, but are also intricately involved in kinase‐mediated signalling pathways and protein complex formation. This coordinated enrichment highlights potential regulatory hubs and molecular targets that may contribute to the pathophysiology of the studied condition. These results provide a foundation for further functional validation and may guide future therapeutic exploration aimed at modulating these critical pathways.

In summary, our study identified hsa‐miR‐15a‐5p as a key differentially expressed miRNA in tuberculosis (TB) infection, with potential regulatory roles in immune modulation and macrophage function. Through target prediction and PPI network analysis, CCND1, CDK6 and CCND2 were highlighted as central genes potentially regulated by miR‐15a‐5p, implicating a post‐transcriptional regulatory axis involving cell cycle control in TB pathogenesis. Enrichment analysis further confirmed that cell cycle regulation, kinase activity and protein complex formation are major biological themes among the predicted targets. These findings not only deepen our understanding of TB‐associated molecular mechanisms but also suggest that miR‐15a‐5p and its downstream targets may serve as potential diagnostic biomarkers or therapeutic targets. Nevertheless, additional experimental validation and clinical studies are required to confirm these interactions and fully elucidate their functional roles in TB infection.

## 5 Conclusion

This study highlights hsa‐miR‐15a‐5p as a key differentially expressed miRNA in active tuberculosis (TB), with potential regulatory roles in immune modulation and macrophage function. Through target prediction and PPI network analysis, CCND1, CDK6 and CCND2 were identified as central genes potentially regulated by miR‐15a‐5p, suggesting a post‐transcriptional axis linked to cell cycle control in TB pathogenesis. Enrichment analyses further underscore the involvement of cell cycle regulation, kinase activity and protein complex formation in TB‐related molecular mechanisms. These findings offer new perspectives on miRNA‐mediated regulation in TB and provide a foundation for future diagnostic and therapeutic exploration.

### 5.1 Limitations

This study's interpretative capacity is constrained by several factors. Initially, the modest cohort size, comprising merely three cases and three controls, potentially restricts the extrapolation of our results. An expansion of the sample size would bolster the validity and reliability of our findings. Second, the conclusions are primarily based on in silico predictions and network analyses; experimental validation of miRNA–target interactions and functional assays are required to confirm the regulatory roles of miR‐15a‐5p and its target genes. Third, although novel miRNAs such as NovelmiRNA‐34/NovelmiRNA‐192 were identified, their biological significance remains speculative and warrants further investigation. Therefore, to overcome these challenges and further clarify the molecular dynamics of TB pathogenesis, additional experimental and clinical inquiries are essential.

## Author Contributions

**Jun Yang:** funding acquisition (lead), project administration (lead), resources (equal), writing – original draft (lead). **Hanliang Dan:** data curation (equal), formal analysis (equal), investigation (lead), project administration (equal), validation (equal). **Maslinda Binti Musa:** project administration (equal), supervision (lead), writing – review and editing (equal). **Yeng Chen:** conceptualization (equal), supervision (equal), writing – review and editing (lead). **Linrong Zou:** formal analysis (equal), validation (equal). **Shanshan Liu:** investigation (equal), methodology (equal), software (equal). **Feng Wang:** data curation (equal), funding acquisition (equal), resources (equal).

## Ethics Statement

All the patients who participated in this study provided written informed consent. The study was carried out according to the principles of the Declaration of Helsinki and was approved by the Ethics Committee of Affiliated Hospital of Guilin Medical University (No. 2023YJSLL‐26).

## Consent

All the authors read and approved the final manuscript.

## Conflicts of Interest

The authors declare no conflicts of interest.

## Data Availability

The RNA sequencing data generated in this study have been deposited in the Gene Expression Omnibus (GEO) database under the accession number [GSE288823]. All other relevant data and materials supporting the findings of this study are available from the corresponding author upon reasonable request.
